# The IBS bug-pain connection

**DOI:** 10.1080/19490976.2024.2308956

**Published:** 2024-02-08

**Authors:** Elena F Verdu, Stephen Vanner

**Affiliations:** Farncombe Family Digestive Health Research Institute, Division of gastroenterology, Department of Medicine, McMaster University, Hamilton, Canada; Division of gastroenterology, Department of Medicine, Queen’s University, Kingston, Canada

In the wake of the opioid crises there has never been a time of greater need for new therapies to treat pain. Conventional opioids are often prescribed yet they cause serious side effects that reduce a patient’s quality of life and drive medical cost.^1^ Moreover, there is a significant risk of addiction and tragically over 80,000 overdoses occur annually in the US.^2^ The opioid crisis has created serious headwinds for pain research, but fortunately other events are propelling the field forward. Notably, the awarding of the Nobel Prize in Physiology or Medicine in 2021 to Drs. David Julius and Ardem Patapoutian for their novel discoveries of the molecular basis of touch and temperature sensations in sensory nerves in the skin, has highlighted exciting advances in the field. Other important discoveries are also occurring, including a growing understanding of the genesis of chronic abdominal pain.

Irritable Bowel Syndrome (IBS), one of the most common disorders of gut–brain interactions, is characterized by chronic abdominal pain associated with altered bowel habits. In the gut, it has been recognized for many years that subtle activation of immune cells, particularly mast cells, leads to release of mediators that can sensitize peripheral afferent nerves or nociceptors.^3^ These sensory nerves become sensitized, leading to exaggerated pain signaling to the brain, called visceral hypersensitivity. A critical unresolved question has been what is causing this subtle immune activation and ensuing visceral hypersensitivity? One of the most exciting discoveries has been that the microbiota are also releasing neuroactive mediators and these mediators can signal through intermediary pathways, such as mast cells, or directly to the nerves to cause visceral hypersensitivity.^4^

In this Collection of Gut Microbes, a series of review and featured articles present accumulating evidence of the role of microbes in the modulation of visceral pain and its implications for the management of abdominal pain in chronic disorders such as IBS. While previous work indicated that microbiota disruption by antibiotics impacts visceral perception and sensory neurotransmitter expression in the gut,^5^ Pujo et al.^6^ elegantly show, using germ-free mice, that absence of gut microbiome leads to increased visceral sensitivity mediated by calcitonin gene-related peptide (CGRP) production.

Four reviews complete this issue and discuss specific microbial mediators and their mechanisms. De Palma et al.^7^ postulate that dietary components can trigger secretion of visceral pain mediators by the bacterial components of microbiota and by host cells. Specifically, fermentable carbohydrates influence histamine-producing bacteria in the gut in a subset of IBS patients, mechanistically supporting the observations that dietary exclusion can benefit patients with irritable bowel syndrome. Caminero et al.^8^ raise the role of bacterial proteolytic imbalance as a new player in the modulation of inflammation, barrier function and pain, postulating a new class of drug development that targets this activity could help restore protease balance and alleviate disease. Van Thiel et al.^9^ remind us that while the bacteriome has been mostly studied in relation to pain, the intestinal mycobiome is emerging as an important player in a subset of IBS hypersensitive patients. Interestingly, their results showed that the visceral hypersensitivity phenotype could be transferred to animal models through fecal transfer and rescued by fungicide treatment.^10^ Shin et al.^11^ point at a gap in clinical translation, where reliable biomarkers for common disorders of gut – brain interaction characterized by abdominal pain, including but not limited to IBS, are needed to develop individualized therapies.

Together these studies highlight that we are rapidly moving toward novel new therapies that target individual or microbial communities and/or pharmacological targets in their signaling pathway. Biomarkers are also emerging that will enable physicians to identify which Bug-Pain disorders are underlying an individual patient’s pain, and thus tailor their therapy in a more personalized fashion to relevant mechanisms rather than simply treating the symptom.
